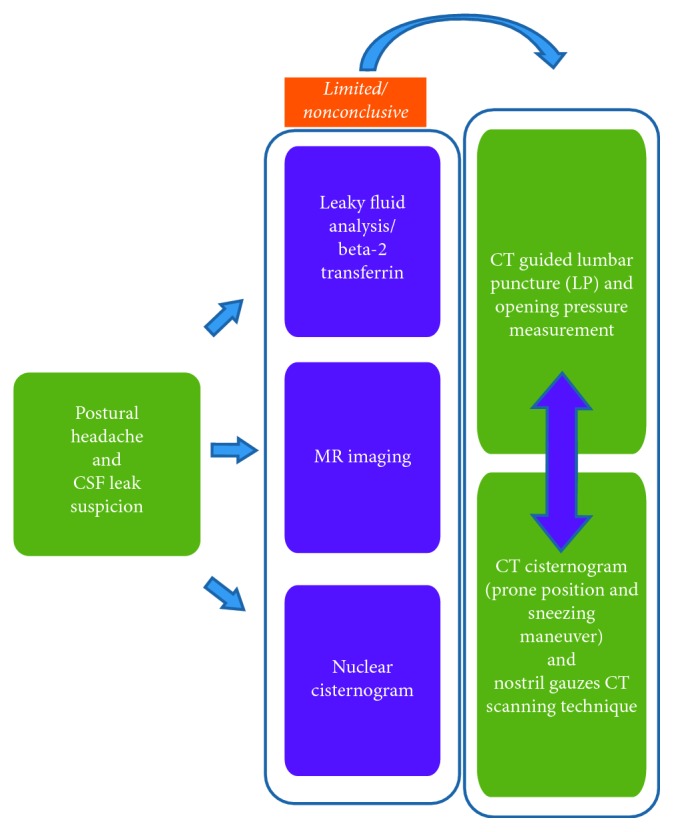# Cryptococcal Meningitis in a HIV Seronegative Patient: A Rare Complication of Cerebrospinal Fluid Leak Detected with a New Cisternographic Technique

**DOI:** 10.1155/2019/3510860

**Published:** 2019-02-20

**Authors:** Chukwunonso Chime, Charbel Ishak, Kishore Kumar, Muhammad Kamal, Srinivasan Krishna, Paul Kelly, Sridhar Chilimuri

**Affiliations:** BronxCare Health System–A Clinical Affiliate of Mount Sinai Hospital and Academic Affiliate of the Icahn School of Medicine, New York, NY, USA

## Abstract

Immune deficiency is usually the underlying predisposing factor for cryptococcal meningitis, though there have been case reports of immunocompetent patients presenting with same. The portal of entry for *Cryptococcus neoformans* is the respiratory tract, and by hematogenous spread, it causes systemic symptoms. The presence of CSF leak is described to have predisposed our immunocompetent patient to infection by this organism possibly through direct spread. The gold standard for diagnosing CSF leak is by cisternography. In this case, we added a technique where nasal gauze is inserted during the procedure and scanned afterwards for dye, thus increasing the confidence of diagnosis of CSF leak through the nares. Prompt diagnosis and treatment is key to prevent adverse outcomes, and we propose that in patients with cryptococcal meningitis without any identifiable risk factor, evaluation for CSF leak should be considered especially with history of head trauma.

## 1. Introduction

Cryptococcosis, an infection caused by the encapsulated yeast *Cryptococcus neoformans* is commonly associated with immunodeficiency states, both human immunodeficiency virus (HIV) and non-HIV related. Cerebrospinal fluid (CSF) leak, although rare, is a well-recognized cause of headache with annual incidence of spontaneous CSF leak estimated to be 5/100,000 [1]. CSF leak occurs as a result of varying etiologies, one of which is trauma, and presence of compromised dura bacteria from the respiratory tract has been known to cause meningitis through a direct spread. Although the respiratory tract is the known portal of entry for *Cryptococcus neoformans*, the organism has not been reported to cause central nervous system (CNS) infections through direct spread. We report to the best of our knowledge, the first known case of CSF leak detected with our new cisternographic technique, predisposing a patient to cryptococcal meningitis in the absence of another immune compromise.

## 2. Case Report

A 50-year-old Hispanic female presented to the emergency room (ER) with complaint of severe headache for 1 day. The headache was described as throbbing with associated photophobia and multiple episodes of nonbloody vomiting. She reported a remote history of closed head trauma and intermittent rhinorrhea, especially on leaning forward, with spontaneous resolution a few months prior to this presentation. Her medical history was significant for intermittent “migraine” and coronary artery disease. Physical examination was notable for photophobia, otherwise no nuchal rigidity or focal motor or sensory neurological deficits; the rest of the examination and vital signs were unremarkable. Laboratory parameters revealed hemoglobin 11.6 g/dl, white cell count (WBC) 13.1 × I0^9^/1 (84.7% neutrophils), serum sodium 138 mEq/l, potassium 4.1 mEq/l, and creatinine 0.7 mg/dl; all liver function tests were within normal limits.

She was admitted to the medical floor where a lumbar puncture was performed with an opening pressure of 35 cm H_2_O, CSF fluid analysis with WBC 1850 cells/uL (segmental 70%), red blood cells 5, protein 175 mg/dl (normal 15–45), glucose 40 mg/dl (normal 40–70), cryptococcal CSF antigen detected with a titer of 1 : 1024 (normal < 1 : 2 titer), negative bacterial antigen, and no growth on bacterial CSF culture. Patient also had a negative HIV serology confirmed by nondetectable HIV ribonucleic acid (RNA) and an absolute CD4 count of 1168.

**Figure 1:**
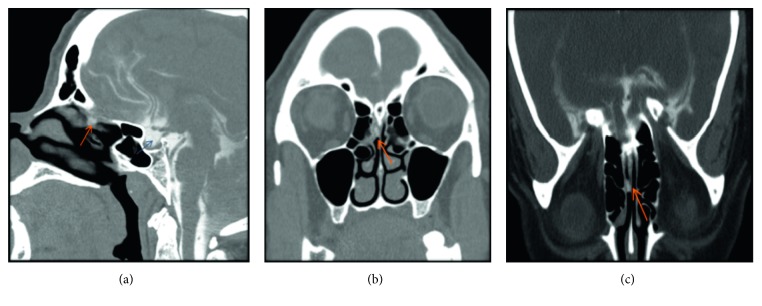
Sagittal (a), axial (b), and coronal (c) images in prone position with scanning during sneezing maneuver. CT cisternogram demonstrated leakage of intrathecally injected contrast (red arrow) noted in the right nasal cavity, along the right aspect of the perpendicular plate of nasal septum, just below the right cribriform plate. Empty sella filled with intrathecally injected contrast (blue arrow).

**Figure 2:**
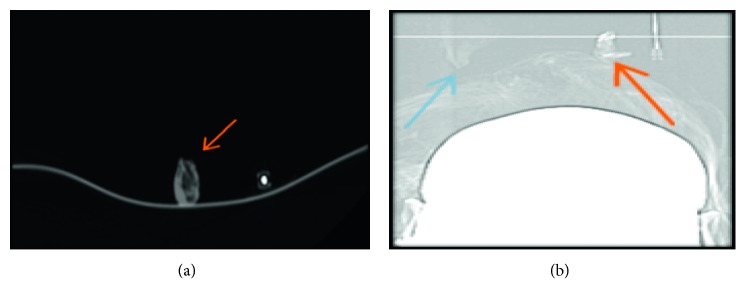
Axial CT scan (a) and scout (b) of gauzes removed from both right (red arrow) and left (blue arrow) nostrils, after being inserted intentionally by the author (C.I.) during real-time cisternogram. This technique confirmed without doubt the presence of leaky contrast in the right nostril, compatible with CSF leakage.

With the patient's history suspicious for traumatic CSF leak, she had a magnetic resonance imaging (MRI) of the head and sinus that were both unrevealing. Given the low sensitivity of a regular MRI in CSF leak diagnosis, a computed tomography (CT) cisternogram was performed. It revealed evidence of leakage of intrathecally injected contrast into the right nasal cavity via the right olfalctory fossa, between the anterior ethmoidal cells and the crista galli bone (Figure 1). This was also confirmed by scanning the gauzes inserted intentionally into both right and left nares by the author (C.I.) during real-time CT cisternogram (Figure 2). This first-time-used innovative, inexpensive, and efficient CT technique confirmed without any doubt the presence of leaked contrast from the opacified CSF in the right nostril gauze when compared to contralateral side; this could have a tremendous role in the preoperative planning, intraoperative performance, and postoperative outcomes.

She was initially started on antimicrobial treatment for presumptive bacterial meningitis and later transitioned to induction therapy for cryptococcal meningitis with liposomal amphotericin B and flucytosine (5-fluorocytosine) after preliminary results of her CSF analysis were obtained. Patient subsequently had an endoscopic-assisted transnasal repair of CSF leak with abdominal fat graft using brainlab navigation system by combined neurosurgical and ear, nose, and throat (ENT) teams. With completion of 2 weeks of induction therapy with liposomal amphotericin B and flucytosine, she demonstrated clinical improvement, and two repeat lumbar punctures during the course of admission showed normalized opening pressure and negative cryptococcal antigen. Fluconazole was then started for consolidation, and she was followed-up on discharge in the outpatient clinic until completion of an 8-week course of fluconazole. Patient has remained symptom free two years after completion of treatment.

## 3. Discussion

Immune compromise is a common underlying predisposition for infection by *Cryptococcus neoformans*, the causative organism of cryptococcosis, with the following immunodeficiency states described in literature: HIV infection, organ transplantation, chronic steroid use, and sarcoidosis [2]. There have been case reports of cryptococcal meningitis in the immunocompetent population and among HIV-negative patients with cryptococcosis; studies have shown no apparent immune deficiency in 10–40% [3]. Immunodeficient states described in HIV-negative cases include nephrotic syndrome, cirrhosis, diabetes, malignancies, and autoimmune diseases [4]. Abbas et al. described a case of cryptococcosis in a HIV-negative patient with history of splenectomy [5]. CSF leak as a predisposing factor for cryptococcal meningitis in a HIV-negative patient has not been described in literature, and for this reason, we feel compelled to present this rare case.

Cryptococcal infections have a wide range of clinical presentations varying from asymptomatic respiratory tract colonization to disseminated infection [6]. Various organs have been described to be affected by cryptococcosis infection, but the most common site is the central nervous system [7]. The respiratory tract is the usual portal of entry of the fungus into the host; following entry, there is a hematogenous spread of the organism to the central nervous system [8]. Unlike other respiratory tract bacteria that have been described in literature to cause direct CNS involvement in the presence of a compromised brain dura, this is the first report of *Cryptococcus neoformans* causing meningitis through this route in an immunocompetent patient. With CNS involvement and meningitis, elevated intracranial pressure, defined as opening pressure of >20 cm H_2_O measured in a reclining position, is present in more than 50% of patients regardless of HIV status [9] and is generally associated with a high CSF yeast burden [10]. Clinical manifestation of cryptococcal meningitis includes fever, nausea, vomiting, and headache; there could also be signs of spinal root irritation in only one-third of patients, commonly the immunocompetent [11]. Other clinical variations exist when comparison is made between this meningitis in HIV and non-HIV infected patients, with the former having much less inflammatory response and shorter duration of symptoms but generally higher burden of organisms [9].

In patients with persistent CSF leakage, meningitis poses a considerable risk and carries a 10% mortality [12] with *Haemophilus influenzae* and *Streptococcus pneumoniae* being the most common pathogens [13]. We are unaware of any published case reports describing *Cryptococcus neoformans* as a causative organism for meningitis in a patient with CSF leak.

For the routine detection of cryptococcal antigens in CSF, certain modalities are available and include historical staining with Indian ink and observation under light microscopy. With almost 100% sensitivity and 98% specificity, newer latex agglutination tests are currently recommended. Definitive diagnosis is made by growing the yeast in CSF culture [14]. We suspect in our case that given the patient was HIV negative and had ongoing drainage of the CSF, the burden of organism may have been low, explaining lack of subsequent growth on CSF culture. Negative CSF culture also raised our concern for possible false-positive CSF cryptococcal antigen latex agglutination test. A few causes have been identified in the literature as potential etiology of false positivity and include cross reaction with *Trichosporon* species infection or disinfectants, soap, or starch [15]. In our index case, there was no clinical evidence of white piedra caused by *Trichosporon species* and no other underling immunocompromise that would predispose her to trichosporonosis; also, no contamination with disinfectants/soap and interference with hydroxyethyl starch were encountered. As part of the initial workup to assess immune status, HIV infection was excluded with negative HIV serologic and RNA testing. Her normal CD4 count also excluded idiopathic T-cell deficiency. Peculiar to our case is a previously unrecognized risk factor for cryptococcal meningitis, a CSF leak, and given her history of traumatic rhinorrhea with postural headache, this was further worked up with a cisternogram to confirm diagnosis and necessitate repair.

Postural headache is an important feature of CSF leak [16]; however, postural headache by itself lacks sensitivity or specificity without a history of head trauma/surgery. Postural headache with rhinorrhea should prompt search for a traumatic CSF leak, as this can lead to life threatening complications in 25–50% of cases including meningitis [16]. In general, the diagnosis of CSF leak would include plain films of the facial bones and skull, CT or MRI cisternography, and high resolution CT. CT cisternography remains the gold standard for diagnosing CSF leak [17]. A history of head trauma and the presence of unusual meningitis could be a result of CSF leak, and we have proposed an algorithm for further diagnostic evaluation (see Figure 3).

Prompt recognition and treatment is required as cryptococcal meningitis is fatal without treatment [18]. The host's immune system and anatomic site of involvement would determine the choice of treatment [19], and with current recommendations, induction therapy is primarily focused at rapid fungicidal activity to achieve sterilization of the CNS [21]. Successful induction regimen evidenced by sterile CSF culture at 2 weeks after initiation of therapy has been linked to favorable outcomes [22]. Regardless of HIV status, an acceptable therapy is induction with amphotericin B and flucytosine for at least 2 weeks, followed by a minimum of 8 weeks of fluconazole for consolidation, but there can be other variations to this regimen [20].

The impact of aggressive management of intracranial pressure in HIV-negative patients is unclear and has not been consistently employed [19]; the same cannot be said for HIV-positive patients. When reduction in intracranial pressure is required, the principal intervention is by lumbar drainage, either by lumbar puncture or use of a lumbar drain [9]. When these options fail to control symptoms of elevated pressure or neurological deficits progress, a ventriculoperitoneal shunt is indicated [9]. CSF leaks are mostly treated either surgically or conservatively; the surgical approach involves transnasal endoscopic, intracranial, and extracranial methods [13]. As cisternogram showed leakage into the right nasal cavity in our patient, she was managed with the transnasal approach by a combined ENT and neurosurgical team. Her symptoms improved with antifungal therapy before discharge and she remained asymptomatic in subsequent clinic follow-up.

## 4. Conclusion

Cryptococcal meningitis is known to be fatal without treatment; prompt recognition is key with regards to changing the clinical course outcome of this deadly disease. Though immune deficiency including HIV has been known as a predisposing factor, there have been reported cases of immunocompetent patients presenting with cryptococcal meningitis. Both spontaneous and head trauma-related CSF leaks are rare conditions, however, when unrecognized, could lead to life-threatening complications. Early recognition is key and could lead to interventions resulting in excellent outcomes. Postural headache, history of trauma/surgery (recent and remote), and the presence of unusual causes of meningitis should prompt workup for CSF leak. CT cisternography remains the gold standard for diagnosing CSF leak, and we propose adding the gauze scanning technique, which is innovative, inexpensive, and efficient.

## Figures and Tables

**Figure 3:**